# Predictive value of von Willebrand factor for venous thrombosis in patients with chronic heart failure complicated with atrial fibrillation after anticoagulant therapy

**DOI:** 10.1186/s12872-023-03167-1

**Published:** 2023-07-13

**Authors:** Jinping Song, Yuan Liu, Guohong Huang

**Affiliations:** Department of Clinical Laboratory, People’s Hospital of Xinjiang Uygur Autonomous Region, No. 91, Tianchi Road, Tianshan District, 830001 Urumqi, P.R. China

**Keywords:** Chronic heart failure, Atrial fibrillation, Venous thrombosis, Von Willebrand factor, Anticoagulant therapy

## Abstract

**Background:**

We investigated the value of von Willebrand factor (vWF) in predicting venous thrombosis in patients with chronic heart failure complicated with atrial fibrillation after anticoagulation therapy.

**Methods:**

Totally, 126 patients with chronic heart failure complicated with atrial fibrillation who were treated with anticoagulant therapy and 60 healthy individuals were enrolled. One year after anticoagulant therapy, venous thrombosis occurred in 19 patients. Clinical data of patients were collected. The plasma vWF activity was detected and compared. The logistic regression analysis was used to analyze the influencing factors of vWF. ROC curve was used to evaluate the predictive value of plasma vWF.

**Results:**

Plasma vWF activity was significantly higher in patients with heart failure and atrial fibrillation than control subjects (*P* < 0.01). The vWF activity in patients with venous thrombosis was significantly higher than that in patients without venous thrombosis (*P* < 0.01). ROC curve analysis showed that the cut-off value of vWF activity for venous thrombosis within one year after anticoagulant therapy was 267.5%, and the AUC was 0.742 (95% CI: 0.764–0.921, *P* < 0.05). The sensitivity was 80.0%, and the specificity was 63.6%. Factors of diabetes, myocardial ischemia, old myocardial infarction, and lower extremity atherosclerosis, but not sex, age, coronary heart disease, hypertension, and cardiac function, had significant effect on vWF activity (*P* < 0.05). Logistic regression analysis showed that vWF activity was significantly related with atherosclerosis of lower limbs and old myocardial infarction, but not significantly related with diabetes and myocardial ischemia. The risk of venous thrombosis in patients with vWF activity greater than 267.5% was 10.667 times higher than that in patients with vWF activity less than 267.5% (*P* < 0.05).

**Conclusion:**

The vWF activity greater than 267.5% has clinical predictive value for the risk of lower extremity venous thrombosis in patients with chronic heart failure complicated with atrial fibrillation within 1 year of anticoagulant therapy.

## Background

Chronic heart failure is a group of clinical syndromes manifested in the terminal stage due to the development of various heart diseases such as coronary heart disease, hypertensive heart disease, cardiomyopathy and valvular heart disease [1]. Heart failure and atrial fibrillation frequently coexist. Clinically, about 40% of patients with chronic heart failure have atrial fibrillation [2, 3]. Epidemiological study suggests that atrial fibrillation is prevalent in 24-44% of patients with acute heart failure, in one third of patients with chronic heart failure, and in more than half (57%) of patients with new-onset heart failure [4]. Meanwhile, atrial fibrillation is a precipitating factor for hospitalization of patients with heart failure, accounting for 19% [5].

Atrial fibrillation is an important risk factor for thrombosis in chronic heart failure [6–8]. Patients with atrial fibrillation complicated by heart failure, even with anticoagulation therapy, have a higher risk of thrombosis than patients without heart failure [1, 9]. It is suggested that oral anticoagulation is reasonable in patients with heart failure and atrial fibrillation, regardless of underlying systolic function, high or low thromboembolic risk score, or the presence of other thromboembolic risk factors [10, 11]. However, anticoagulation in patients with heart failure and atrial fibrillation is still suboptimal [12].

Endothelial cell injury and endothelial dysfunction are common in patients with heart failure. Under physiological conditions, intact endothelial cells can express various anticoagulant factors, such as tissue factor pathway inhibitor, thrombomodulin, endothelial cell protein C receptor and heparin like proteoglycan, to prevent thrombosis [13]. Endothelial dysfunction, which can lead to the imbalance of procoagulant and anticoagulant factors and recruitment of other blood cells, is the main factor leading to venous thrombosis [14, 15]. Von Willebrand factor (vWF) is mainly synthesized and secreted by endothelial cells and plays an important role in thrombosis. The activated endothelial cell can release Weibel-Parade bodies containing vWF, P-selectin and other procoagulant and proinflammatory components (cytokines and chemokines). Platelet GPIb α interacts with the pre exposed A1 domain of vWF polymers to promote thrombosis [16–19]. P-selectin can recruit neutrophils to form neutrophil extracellular traps, which can interact with vWF and promote venous thrombosis. For example, neutrophil extracellular traps directly interact with vWF through electrostatic force, and this interaction keeps NETs on the endothelial surface [17]. High plasma concentration of vWF is considered to be a hallmark of endothelial dysfunction, a predisposing state of atherosclerosis and the pathological basis of thrombosis [20, 21]. Elevated vWF levels are associated with an increased risk of thromboembolism and cardiovascular disease [22, 23]. It has been shown that patients with cardiovascular disease, especially patients with atrial fibrillation, have elevated vWF concentrations [24, 25]. In our previous study, we also found that the activities of vWF and coagulation factor VIII in patients with chronic heart failure complicated by thrombosis were significantly higher than those in patients without thrombosis [26]. Anticoagulant therapy for patients with atrial fibrillation has become the primary strategy to prevent thrombosis. However, due to risk factors such as coronary heart disease, hypertension, diabetes, lower extremity atherosclerosis, and advanced age in patients with heart failure, as well as individual differences in the anticoagulant warfarin, and compromised effects of dabigatran and rivaroxaban by liver and kidney dysfunction, patients with heart failure and atrial fibrillation often experience thromboembolism under the condition of anticoagulation therapy [27]. Therefore, it is necessary to evaluate the efficacy of anticoagulation in patients with heart failure and atrial fibrillation.

Herein, we explored the role of plasma vWF in predicting the risk of venous thrombosis during anticoagulation therapy in patients with heart failure and atrial fibrillation. Our findings may provide evidence for prediction and prevention of venous thrombosis in such patients.

## Methods

### Study participants

We enrolled 126 patients with chronic heart failure and atrial fibrillation who were treated from January 2017 to October 2020. There were 74 males and 52 females, with an average age of 74.15 ± 7.89 years. Among them, 39 patients received warfarin anticoagulant therapy, 87 patients received anticoagulant therapy with new oral anticoagulant drugs (rivaroxaban or dabigatran). The initial dose of warfarin was 2.5 mg per day, for 3–5 days. Then, the dose of warfarin was adjusted according to the prothrombin time/international normalized ratio to maintain the international normalized ratio at 2–3. Rivaroxaban (10 mg) and dabigatran (110 mg) were orally given once daily. Exclusion criteria: (1) patients with family history of hereditary thrombosis; autoimmune diseases, platelet functional diseases, bleeding/coagulation disorders, acute inflammation, systemic inflammatory response syndrome, severe liver and kidney damage, tumors, pregnancy, etc. were excluded; (2) patients received surgery in the past six months were excluded; (3) patients with fracture, trauma, cerebral hemorrhage, or acute myocardial infarction were excluded. The patients were followed up by telephone for one year, with the occurrence of venous thrombosis as the end event. Peripheral blood was collected from patients at one year after anticoagulant therapy. For control, 60 healthy individuals (including 30 males and 30 females, with an average age of 70.23 ± 6.99 years old) undergoing physical examination during the same period were enrolled. This study was approved by the Ethics Committee of People’s Hospital of Xinjiang Uygur Autonomous Region. All methods were performed in accordance with the Declaration of Helsinki. All subjects signed the written informed consent.

### Data collection

Clinical data of patients, such as age, gender, medical history, and treatment, were collected.

### Detection of biochemical indexes

The plasma vWF activity and D-dimer (D-DI) concentration were determined by STAGO automatic coagulation analyzer with the immunoturbidimetric method. The B-type natriuretic peptide (BNP) concentration was determined using the Triage BNP assay (Biosite, San Diego, CA, USA) based on immunofluorescence.

### Statistical methods

All data were processed by SPSS 19.0. The measurement data are expressed as mean ± standard deviation and were compared with one-way analysis of variance or independent-sample t test. Count data is expressed as rate, and was analyzed with the χ2 test. Logistic regression was used to analyze the relationship between vWF and various clinical factors. ROC curve analysis was conducted to evaluate the performance of vWF activity in predicting venous thrombosis in patients with chronic heart failure and atrial fibrillation. P < 0.05 indicates statistically significant difference.

## Results

### Basic clinical data of subjects

The basic clinical data of patients is shown in Table 1. During the follow-up period, 19 cases of lower limb venous thrombosis occurred, including 14 cases of intermuscular venous thrombosis, 1 case of popliteal vein thrombosis, 1 case of small saphenous vein thrombosis, 1 case of femoral vein thrombosis, 1 case of deep vein thrombosis, and 1 case of posterior tibial vein thrombosis. Among these 19 cases, there were 5 cases taking warfarin and 14 cases taking new oral anticoagulants (rivaroxaban or dabigatran). The χ2 test showed that there was no significant difference in thrombosis between patients taking warfarin and those taking new oral anticoagulants (*P* < 0.05).

**Table 1:** Basic information of patients

Age (years)	74.15 ± 7.89[41–93]
Male, n (%)	74(59%)
Hypertension	68(54%)
Coronary heart disease	70(56%)
Diabetes	44(35%)
Old myocardial infarction	41(33%)
Myocardial ischemia	46(37%)
Lower extremity atherosclerosis	58(46%)
Cardiac function classification	
Class III	56(44%)
Class IV	70(56%)
Lower extremity venous thrombosis	19(15%)
Interstitial venous thrombosis	14(74%)
Popliteal vein thrombosis	1(5%)
Femoral vein thrombosis	1(5%)
Deep vein thrombosis	1(5%)
Small saphenous vein thrombosis	1(5%)
Posterior tibial vein thrombosis	1(5%)
Anticoagulation therapy	
Warfarin	39(31%)
New oral anticoagulants (rivaroxaban or dabigatran)	87(69%)
Laboratory data	
BNP (pg/L)	1317.47 ± 929.09
D-DI (µg/L)	1.74 ± 1.37
vWF (%)	250.58 ± 88.05

Note: D-DI, D-dimer; BNP, B-type natriuretic peptide; vWF, von Willebrand factor

### Comparison of plasma vWF activity

As shown in Table 2, the plasma vWF activity in patients with heart failure and atrial fibrillation after anticoagulation therapy was significantly higher than that of the normal control group (P < 0.01). The activity of vWF in patients with venous thrombosis was significantly higher than that in those without venous thrombosis (P < 0.01). However, the vWF activity was not significantly different between patients taking warfarin and those taking new oral anticoagulants.

**Table 2:** Comparison of plasma vWF activity

	Number of cases	vWF (%)
Normal control	60	91.34 ± 21.53
Patients with heart failure and atrial fibrillation	126	248.53 ± 107.19^*^
Patients with heart failure and atrial fibrillation without thrombosis	107	237.51 ± 113.45^*^
Patients with heart failure and atrial fibrillation with thrombosis	19	277.22 ± 101.74^*Δ^
Patients taking warfarin	39	244.34 ± 89.81
Patients taking new oral anticoagulants (rivaroxaban or dabigatran)	87	249.281 ± 102.00

Note: Compared with control, ^*^P < 0.05; Compared with patients without thrombosis, ^Δ^*P* < 0.05

### Predictive analysis of plasma vWF activity on venous thrombosis after anticoagulant therapy in patients with heart failure and atrial fibrillation

**Fig. 1:**
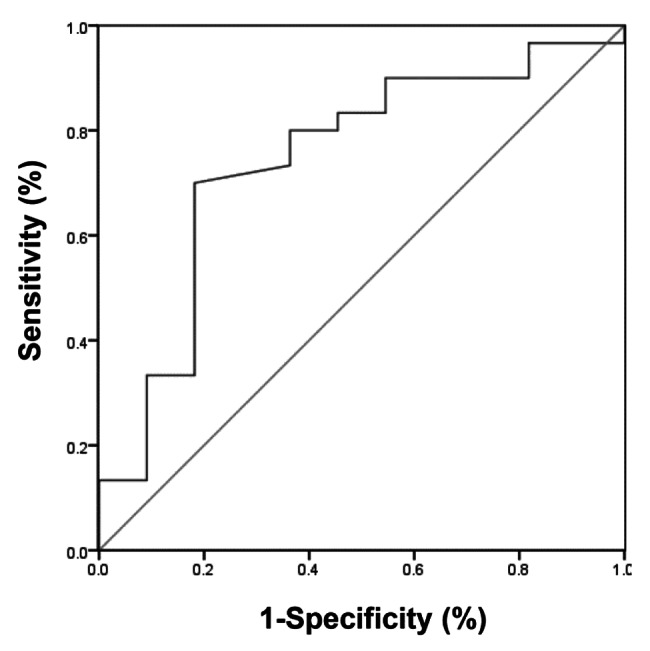
**ROC curve of vWF.** Predictive effect of plasma vWF activity on venous thrombosis after anticoagulant therapy in patients with heart failure and atrial fibrillation was evaluated by ROC curve. The sensitivity and specificity were shown

The ROC curve was used to analyze the predictive value of vWF activity for the occurrence of venous thrombosis within one year in patients with heart failure and atrial fibrillation receiving anticoagulant therapy. As shown in Fig. 1, the area under the curve for vWF was 0.742 (95%CI: 0.764–0.921, P < 0.05). The cut-off value of vWF was 267.5%, with sensitivity of 80.0%, and specificity of 63.6%.

### Relationship of plasma vWF activity with clinical factors in patients with heart failure and atrial fibrillation after anticoagulation therapy

As shown in Table 3, patients with different vWF activities were not significantly different in sex, age, coronary heart disease, hypertension, and cardiac function classification as well as BNP and D-DI concentrations (P > 0.05). However, they showed significant differences in diabetes, myocardial ischemia, old myocardial infarction, and, atherosclerosis of the lower extremities (P < 0.05).

**Table 3:** Relationship of plasma vWF activity with clinical factors in patients with heart failure and atrial fibrillation after anticoagulation therapy

Clinical factors	Number of cases	vWF activity (%)			
		< 267.5%	≥ 267.5%	*χ*^2^/*t*	*P*
**Sex**					
Male	74	44	30	0.039	0.843
Female	52	30	22		
**Age**					
< 70 years	54	30	24	0.096	0.757
≥ 70 years	72	38	34		
**Coronary heart disease**					
Yes	70	32	38	2.806	0.094
No	56	34	22		
**Hypertension**					
Yes	68	46	22	0.002	0.961
No	58	39	19		
**Diabetes**					
Yes	44	18	26	5.237	0.002
No	82	51	31		
**Myocardial ischemia**					
Yes	46	16	30	11.674	0.001
No	80	53	27		
**Old myocardial infarction**					
Yes	41	14	27	12.181	0.000
No	85	57	28		
**Atherosclerosis of the lower extremities**					
Yes	58	12	46	43.273	0.000
No	68	54	14		
**Cardiac function classification**					
III	56	26	30	0.514	0.473
IV	70	37	33		
**BNP (pg/L)**	86/40	1162.83 ± 759.38	1480.47 ± 1092.20	-0.944	0.358
**D-DI (µg/L)**	79/47	1.49 ± 1.16	2.27 ± 1.65	-1.421	0.167

Note: Von Willebrand factor (vWF); B-type natriuretic peptide (BNP); D-dimer (D-DI)

### Regression analysis of vWF activity and clinical factors

The clinical factors with significant differences were used as independent variables to conduct a binary regression analysis. The relationship between vWF and various clinical factors was analyzed. The results showed that vWF activity was significantly related to lower extremity atherosclerosis and old myocardial infarction (P < 0.05), but not significantly related to diabetes and myocardial ischemia (P > 0.05) (Table 4).

**Table 4:** Binary regression analysis of vWF activity and clinical factors

Clinical factors	*B*	*OR*	*95% CI*	*P*
Lower extremity atherosclerosis	2.161	8.677	1.339–56.325	0.023
Diabetes	0.612	1.845	0.319–10.668	0.494
Myocardial ischemia	1.691	5.424	0.408–72.014	0.200
Old myocardial infarction	2.151	8.590	1.492–49.438	0.016

### Regression analysis of vWF activity in predicting venous thrombosis during anticoagulation treatment in patients with chronic heart failure and atrial fibrillation

Using the cut-off value of vWF activity (267.5%) as the independent variable, and thrombosis occurrence as the dependent variable, logistic binary regression analysis was performed to evaluate the risk of vWF activity on the occurrence of lower extremity venous thrombosis in patients with chronic heart failure and atrial fibrillation during anticoagulation therapy. The results showed that the risk of lower limb venous thrombosis in patients with vWF activity greater than 267.5% was 10.667 times that of vWF activity less than 267.5% (P < 0.05) (Table 5).

**Table 5:** Regression analysis of vWF activity in predicting venous thrombosis during anticoagulation treatment in patients with chronic heart failure and atrial fibrillation

Variable	*B*	*Exp (B)*	*95% CI*	*P*
vWF activity ≥ 267.5%	2.367	10.667	1.894–60.078	0.007

## Discussion

Chronic heart failure and atrial fibrillation are independent risk factors for thrombosis, and the incidence of thrombotic events in patients with heart failure and atrial fibrillation is higher [28, 29]. Thrombosis is closely related to the survival rate and prognosis of patients with heart failure. Therefore, actively preventing thrombosis can help improve the survival rate and quality of life of patients with heart failure. Antithrombotic and anticoagulant therapy has been the consensus for effective prevention of thrombosis in patients with atrial fibrillation. However, currently, anticoagulant therapy for patients with atrial fibrillation and patients with atrial fibrillation combined with other cardiovascular diseases is seriously insufficient [30, 31]. Meanwhile, patients receiving anticoagulation therapy have different anticoagulation effects due to differences in liver and kidney function, age, course of disease, underlying etiology, and dosage of medications [32–34]. Therefore, effective identification and timely diagnosis of thrombosis in patients receiving anticoagulation therapy is particularly important.

In this study, 19 of the 126 patients with heart failure and atrial fibrillation who received anticoagulation therapy had lower extremity venous thrombosis. Among them, 1 case had lower extremity deep venous thrombosis, but most of them had lower extremity intermuscular venous thrombosis. The incidence of venous thrombosis was 15.08%. This rate was higher than that reported by Zhang et al. (4.69%) [34], but lower than that reported by Wu et al. (25%) [35]. The first possible reason may be the different follow-up time. The follow-up time of this study was 1 year, in the study by Zhang et al. was 3 months [34], and, in the report by Wu et al. [35] was 9 months. The second possible reason may be the age differences in the study subjects. Age is a risk factor for many diseases, including heart failure and atrial fibrillation [35].

The vWF is a marker of damaged vascular endothelial cells and its activity is directly proportional to the degree of damage [36]. The activity of vWF is affected by a variety of clinical factors, including heart failure, myocardial infarction, coronary heart disease, hypertension, diabetes, vascular diseases, blood diseases, tumors and infections [37–41]. For example, the drug carvedilol is reported to significantly reduce vWF activity [42]. This study found that vWF activity was related to diabetes, myocardial ischemia, old myocardial infarction, lower extremity atherosclerosis, and had a positive regression relationship with lower extremity atherosclerosis and old myocardial infarction. This suggests that in patients with heart failure and atrial fibrillation, vascular damage may still be the main factor leading to lower extremity venous thrombosis during the period of anticoagulation therapy.

Rivarxaban and apixaban are direct FXa inhibitors. Previous study has shown that rivaroxaban can protect and repair endothelial cells [43]. It is worth discussing whether rivarxaban or apixaban can affect vWF activity and thereby affect blood coagulation. In our study, we found no significant difference in vWF activity between patients taking warfarin and those taking new oral anticoagulants. Consistently, Schultz et al. studied the effect of new anticoagulants on vWF activity and antigen during the treatment of patients with venous thrombosis and found that rivaroxaban had no significant effect on vWF antigen and activity [44]. In the comparative study of the effects of apixaban and warfarin on coagulation markers in patients with atrial fibrillation, it was confirmed that there was no significant difference in the vWF antigen levels at 2 months between the apixaban and warfarin groups [45]. Although rivaroxaban has protective and repair effects on endothelial cells, the relationship between these repair effects and plasma vWF activity have not been observed. Therefore, further studies are needed to explore the effects of warfarin or direct FXa inhibitors on endothelial cell function and vWF activity.

The predictive value of vWF in cardiovascular and thrombotic diseases has gradually been confirmed [46–48]. Wang et al. reported that the plasma vWF activity of patients in thrombotic disease group was higher than that in non-thrombotic group, and its specificity and sensitivity for predicting thrombotic disease were 78.85% and 76.19%, respectively [49]. In the present study, the ROC analysis showed that the specificity and sensitivity of vWF for predicting lower extremity venous thrombosis in patients with heart failure and atrial fibrillation were 63.6% and 80.0%, respectively. The reason for the low specificity may be that both heart failure and atrial fibrillation are high-risk factors of thrombosis, and the vWF activity in such patients is generally increased. These findings suggest that vWF has a certain ability to predict venous thrombosis in patients with heart failure and atrial fibrillation during the period of anticoagulation therapy.

Chronic heart failure is the terminal stage of cardiovascular disease, which is combined with many basic diseases and is with more complicated conditions. Clinical study has confirmed that the incidence of thrombosis in elderly patients with atrial fibrillation and heart failure after anticoagulation therapy was significantly lower than that of untreated patients [50]. Patients with heart failure and atrial fibrillation should be given anticoagulation therapy according to their thrombus score when they are without contraindications to anticoagulation. However, recommendations for anticoagulation therapy for patients with heart failure and atrial fibrillation are currently not available. Therefore, further studies are needed. This study found that 15.08% of patients with chronic heart failure and atrial fibrillation had lower extremity venous thrombosis under the condition of anticoagulation therapy, suggesting that anticoagulation therapy cannot reverse the coagulation disorder that has occurred, and that there may be also insufficient anticoagulant therapy and monitoring for this type of patient.

This study has some limitations. First, the number of patients was small. Second, blood type was not measured. Patients with blood type O have lower circulating VWF levels, which may affect the results. Third, no scheduled follow-up was scheduled. Further studies are warranted.

## Conclusion

In summary, we demonstrate that the vWF activity greater than 267.5% has clinical predictive value for the risk of lower extremity venous thrombosis in patients with chronic heart failure complicated with atrial fibrillation within 1 year of anticoagulant therapy. Our findings suggest that vWF may help to indicate the occurrence of venous thrombosis in patients with heart failure and atrial fibrillation.

## Data Availability

The datasets used and/or analysed during the current study are available from the corresponding author on reasonable request.

## List of abbreviations

vWF: Von Willebrand factor
BNP: B-type natriuretic peptide
D-DI: D-dimer
